# Effective Evaluation of Nursing Intervention Based on Pediatric Early Warning Score for Pediatric Patients in the Respiratory Department

**DOI:** 10.1155/2022/3769851

**Published:** 2022-09-19

**Authors:** Lihua Huang, Lemei Cheng, Yixia Sun, Fuhui Nian, Tingyue Tao, Jinbo Wu, Haiqiang Ye

**Affiliations:** ^1^Pneumology Department, Anhui Provincial Children's Hospital, Hefei 230051, China; ^2^Department of Quality Control, Anhui Provincial Children's Hospital, Hefei 230051, China

## Abstract

**Objective:**

This study evaluated the impact of nursing intervention based on the Pediatric Early Warning Score (PEWS) on pediatric patients.

**Methods:**

A retrospective analysis was performed on 120 children admitted to the pediatric respiratory unit of Anhui Children's Hospital, and they were randomly and equally assigned to the observation group (PEWS-based nursing intervention) and the control group (usual care). The following parameters were compared: incidence of unplanned admissions to the pediatric intensive care unit (PICU), disease progression, average hospitalization days, hospital costs, time required for nursing assessment and management, nursing record error rate, and medical satisfaction.

**Results:**

The incidence of unplanned admissions to PICU and disease exacerbations, mean hospital days and hospital costs were significantly lower in the observation group than in the control group (*P* < 0.05). Compared with the control group, the observation group had lower nursing care time, time to assess the disease, and an error rate in writing the entire nursing record. In addition, the accuracy rate of nurse assessment and the satisfaction rate of doctors and nurses in the observation group were significantly higher than those in the control group (*P* < 0.05).

**Conclusion:**

The PEWS-based nursing intervention not only reduced the unplanned admission rate, the incidence of disease worsening, and the average number of hospital days and hospital costs in the PICU but also accelerated the recovery process of pediatric patients in the respiratory unit. Meanwhile, PEWS-based nursing interventions can effectively improve nursing efficiency and medical care satisfaction.

## 1. Introduction

In children, the respiratory system is not fully developed, the airways are narrow, the number of alveoli is less, the ability to regulate breathing is poor, and infections are more likely to cause airway obstruction and lead to respiratory failure [1]. Although children have better compensatory mechanisms than adults, when their compensatory capacity is overwhelmed, their disease deteriorates almost immediately, severely affecting their survival and prognosis [1, 2]. Disease progression and death in hospitalized children is predictable and preventable, and clinical deterioration usually occurs before 24 hours of physical change. The care and treatment of critically ill children in respiratory medicine is a particular challenge, and the difficulty in identifying the severity of disease and the inability to correctly measure and interpret signs is a current challenge in this field [3]. Therefore, it is important to systematically assess the child, highlight the characteristics of nursing care for the respiratory child, and identify the critically ill child in a timely manner [4]. Whether in the emergency room or in the inpatient/general ward, many patients, especially pediatric patients, have more unpredictable disease transitions that can change or progress at any time. Therefore, early identification of patients with critical or potentially deteriorating conditions, early triage, and timely intervention are necessary. However, at present, the judgment of the degree of criticality of patients attending medical institutions depends largely on personal subjective experience and intuition, resulting in the inability to correctly identify and timely handle critically ill/potentially critically ill patients, resulting in medical pitfalls and even disputes. Therefore, there is a need to use some objective criteria for early warning of health care workers and timely detection of critical illnesses or potentially critical illnesses in clinical work. Various assessment methods have been applied in clinical practice, and those commonly used at home and abroad include the Acute Physiology and Chronic Health Score (APACHE), simplified acute physiological parameters score (SASP), and the probability of death model (MPM). However, these scoring systems test numerous items, take a long time to perform, are difficult to assess and intervene quickly in patients, and are hardly applicable to pediatric patients. The Pediatric Early Warning Score (PEWS) is a scoring system that identifies clinical deterioration in children. It uses bedside parameters for scoring, does not require complex and expensive equipment, is simple to perform, easy to implement, and is objective and efficient [5]. It is widely used abroad for early warning and monitoring of critically ill or potentially critically ill patients in the emergency room or in the inpatient/general ward, while it is still in an almost blank state in China. This study intends to analyze whether PEWS-based interventions can improve the care of children in respiratory units.

## 2. Research Methodology

### 2.1. Clinical Data

A total of 120 children, including 62 males and 58 females, who were diagnosed and treated in the Department of Respiratory Medicine of Anhui Children's Hospital from January to December 2021 were equally and randomly assigned into the observation group and control group based on the method of random number table. Inclusion criteria: ① age <14 years old; ② first hospitalization in the respiratory department of our hospital; ③ complete clinical data. Exclusion criteria: ① this score does not include the assessment of delivery, so neonates aged 0–28 days are excluded; ② children who are transferred to the pediatric intensive care unit (PICU) within 24 hours admission; ③ children or family members cannot cooperate to complete the experiment. This study has been approved by the ethics committee of our hospital, and informed consent was obtained.

### 2.2. Intervention Methods

The control group received routine care and disease observation: monitoring the child's body temperature, blood pressure, heart rate, and respiration; recording the child's consciousness, changes in the skin, oral cavity, and eyelid mucosa; and keeping the oral cavity clean. When the child's physiological indicators were abnormal, the nurse reported it to the doctor, who issued a medical prescription that the nurse carried out. All medical staff in the observation group attended a 2-hour training session before implementation. Based on the control group, the observation group identified and resuscitated children with deteriorating conditions according to PEWS, as shown in Table 1. The PEWS consisted of three dimensions: behavioral awareness, cardiovascular system, and respiratory system. It provides reliable information for clinical nurses to quickly, objectively, and accurately assess the condition of children [6]^.^ Each dimension is assigned a score of 0 to 3, and PEWS is the sum of the scores of the three dimensions. The higher the points, the more serious the disease [7]. PEWS total score 0–1 points: no treatment, continue to observe; 2 points: notify the responsible nurse to use PEWS for continuous monitoring, consider whether there are symptoms such as pain and fever, and calculate the fluid balance and urine output; 3 points: evaluate at least every 24 hours, based on 2 points, dynamically evaluate, observe the child and notify the specialist nurse; 4 points: evaluate at least every 8 hours, notify the doctor or resident doctor on duty, and prepare for transfer; >4 points, score increase >2 points, single score 3 points: The evaluation is performed every 4 hours, and the general inpatient and PICU doctors are notified to arrive at the scene within 15 minutes, cooperate with the rescue, and prepare for the transfer.

### 2.3. Evaluation Indicators

Children's clinical variables and demographic data were obtained from records. The incidence rates of unplanned admission to PICU and disease deterioration, average hospitalization days and hospitalization expenses, medical and nursing satisfaction, and error rate of nursing records were assessed.

Unplanned admission to the PICU was defined as complications during hospitalization in the general ward of the child, who was transferred to the PICU for further treatment after consultation with an intensive physician.

Condition deterioration: ① Need mechanical ventilation, including noninvasive ventilation, endotracheal intubation, or tracheostomy; ② Use vasoactive drugs, including dopamine, dobutamine, epinephrine, norepinephrine, and isoproterenol or milrinone, etc.; ③ perform cardiopulmonary resuscitation or start extracorporeal membrane oxygenation [8].

Nursing job satisfaction. A homemade questionnaire was used to investigate the satisfaction of nurses and physicians with nursing care. The questionnaire included three dimensions: whether the nurses' observation, judgment, and reporting of the child's condition were timely and accurate; whether the treatment was appropriate; and whether the medical and nursing cooperation was satisfactory. Each dimension was divided into 0: very dissatisfied, 1: dissatisfied, 2: average, 3: satisfied, and 4: very satisfied. Higher scores (ranging from 0–12) indicate higher satisfaction.

The time to assess the child's condition is the time from the time the nurse detects a change in the child's condition to the time of accurate assessment of the child's condition. Nursing time was defined as the time it took for the nurse to perform nursing actions on the child after assessing his or her condition. The accuracy of the assessment was defined as the nurse's decision at the bedside. Whether the assessment was consistent with the final medical and nursing records. The evaluation of nursing record writing included, among other things, that the frequency of recording met the requirements, that the treatment could be accurately recorded according to medical orders, and that the actual condition of the child could be reflected in detail. The condition of the child and the nursing measures are consistent with the current situation. The nursing records reflect the characteristics of the children in the respiratory department. The sentences are fluent and correctly expressed without typos.

### 2.4. Statistical Method

SPSS 25.0 software processed data which were displayed as mean ± standard deviation (SD) or the percentage and assessed by *t*-test or chi-square test. *P* < 0.05 indicated a significance.

## 3. Results

### 3.1. Patients Characteristics

As shown in Table 2, 6 cases out of 120 children dropped out, including 4 cases in the control group and 2 cases in the observation group. Finally, 114 cases were enrolled, including 60 males and 54 females, aged 1–14 years (mean age: 7.17 ± 3.09). There were 58 cases in the observation group and 56 cases in the control group. No significant differences were found between the two groups in terms of age, weight, and gender (*P* > 0.05).

### 3.2. Comparison of Hospitalization between the Two Groups

As shown in Table 3, the incidence of unplanned PICU admissions and disease exacerbations, mean hospital costs, and mean hospital days were significantly lower in the observation group than in the control group (*P* < 0.05).

### 3.3. Comparison of Processing Time and Satisfaction between Two Groups

As shown in Table 4, the observation group had significantly lower nursing care time and disease assessment time, and higher assessment accuracy and physician-nurse satisfaction than the control group (*P* < 0.05).

### 3.4. Comparison of Writing Error Rate of Nursing Records

As shown in Table 5, the observation group had a significantly lower frequency mismatch, time not recorded, inconsistent clinical practice, difficulty in reflecting specialty characteristics, type and misrepresentation than the control group (*P* < 0.05).

## 4. Discussion

In clinical work, early and timely assessment of children's conditions is very important. The Pediatric Critical Care Score (PCIS) is a commonly used method to assess the condition of critically ill children in China, but the score includes up to 10 physiological indicators such as electrolytes and liver and kidney function, which are difficult to apply easily and quickly in clinical work [9]. PEWS typically takes routine physiological measures such as vital signs and laboratory data as input and assesses the risk of clinical deterioration events in children as output [10]. In China, the PEWS score is mainly used in emergency triage as well as in clinical assessment applications in pediatric neurosurgery, respiratory medicine, neurology, and hematology [11–14]. When a child's score exceeds a certain threshold, the nurse alerts the appropriate clinician for further evaluation and intervention. Thus, as a relatively well-established predictive and assessment tool, the PEWS provides an accurate picture of disease prognosis, outcomes, and other indicators. It is suggested that PEWS be widely used as an important part of the assessment of children's condition in pediatric wards [15].

Currently, in clinical work in general pediatric wards, communication between physicians and nurses about diseases is mostly subjective and inaccurate expressions such as “mild, moderate, or severe” [12]. The junior nurses have little clinical experience and insufficient ability to observe the disease, assess dynamically, analyze and solve problems actively [13]. In addition, children's conditions are hidden and change rapidly, and they often can only mechanically carry out medical orders [16, 17]. Using PEWS can improve the communication efficiency of clinical doctors and nurses, especially the independence and self-confidence of junior nurses. PEWS standardizes and quantifies children's abnormal physiological indicators, which helps medical staff to easily and quickly identify and treat critically ill or potentially critically ill children, facilitates rational allocation of medical resources, and improves the safety of children. Based on the above analysis, this study used children hospitalized in pediatric general wards as the study subjects for early identification and early intervention of critically or potentially critically ill patients. The study showed that the PICU transfer rate, the incidence of disease deterioration, average length of stay, and hospitalization cost of children hospitalized in pediatric general wards were significantly reduced after intervention with the PEWS graded response strategy, as well as higher satisfaction of children's families and physicians, and the literature was consistent with our results [18]. The reduction in nursing intervention time and disease assessment time, the increase in assessment accuracy, and the increase in nursing satisfaction after the PEWS-based intervention in this study indicate that the PEWS-based nursing documentation method can identify children's problems in a timely manner and provide effective care in the first instance. It reduced the time to assess and care for the child's condition, especially for junior nurses, and improved efficiency and avoided delays in treatment due to excessive time.

Nursing documents are medical documents with legal benefits, and they must be written in strict accordance with document writing standards. In this study, after the implementation of the nursing intervention, all nurses were able to write nursing records in a timely manner and in accordance with the frequency of medical orders and also in line with clinical reality, and the overall nursing record writing error rate decreased significantly. This method improved the timeliness, objectivity, and accuracy of nursing documentation and reduced the work pressure of nurses. There was still one case of specificity and misspellings and expression errors in the observation group. This may be due to the fact that the intervention time is still short and individual nurses have not yet mastered the issues. More centers and a large-scale nursing intervention model based on PEWS are needed in the future. These samples were further validated by the experiment.

## 5. Conclusion

PEWS-based pediatric interventions can not only reduce the number of unplanned PICU admissions, the incidence of exacerbations, the average length of stay, and hospital costs, and accelerate the recovery process of children, but also effectively improve the efficiency of care and medical satisfaction. The main problems faced by pediatric acute and critical care workers now are how to find the most suitable PEWS system and standardize it so that it can be widely accepted; how to implement the PEWS system more effectively and train the corresponding medical and nursing staffs effectively; how to measure those important vital indicators of patients more accurately and record and calculate the scores more systematically. Through unremitting exploration and research, we believe that a unified and more effective PEWS system can be applied in clinical work in the near future.

## Figures and Tables

**Table 1:** PEWS scoring criteria (points).

Index	0	1	2	3
Conscious state	Normal	Lethargic	Developed irritations	Lethargy/coma decreased response to pain
Cardiovascular system	Skin tone pink, CRT1-2s	Pale complexion, CRT3 s	Gray complexion, CRT4 s Heart rate 20 beats/min higher than normal	Gray complexion, clammy skin CRT ≧5 s Heart rate 30 beats/min higher than normal or bradycardia
Respiratory system	Normal range without respiratory issues	Respiratory rate is higher than normal, increased by 10 beats/min, FiO_2_ 0.3 or inspiratory flow 4 L/min	Respiratory rate is higher than normal, increased by 20 beats/min FiO_2_ 0.4 or inspired oxygen flow 4 L/min	Respiratory rate decreased by 5 beats/min compared to normal, Moaning with sternal inspiratory depression, FiO_2_ 0.5 Inhaled oxygen flow 8 L/min

**Table 2:** Patients characteristics.

Group	Number	Age range ($\bar{X}\pm s$, years)	weight ($\bar{X}\pm s$, kg)	Gender ratio (male/female)
Control group	56	3.32 ± 2.72	15.56 ± 8.08	30/26
Observation group	58	4.33 ± 3.24	18.39 ± 9.97	30/28
*t/χ* ^*2*^		1.795	1.669	0.039
*P*		0.075	0.098	0.843

**Table 3:** Comparison of complications and hospitalization of children.

Group		Admission to PICU [*n* (%)]	Worsening condition [*n* (%)]	Average hospital costs ($\bar{X}\pm s$m Yuan)	Mean hospital stay (±*S*, days)
Control group	56	9 (16.07)	13 (23.21)	10484.20 ± 1342.02	10.11 ± 2.36
Observation group	58	2 (3.45)	5 (8.62)	9052.02 ± 3574.78	7.90 ± 2.06
*t/χ* ^*2*^		5.207	4.564	−2.850	−5.318
*P*		0.022	0.033	0.006	0.000

**Table 4:** Comparison of processing time and satisfaction.

Group		Nursing time ($\bar{X}\pm S$, min)	Illness assessment time ($\bar{X}\pm S$, min)	Accuracy of evaluation [*n* (%)]	Doctor satisfaction ($\bar{X}\pm S$, points)	Nurse satisfaction (±*S*, points)
Control	56	7.61 ± 2.23	7.60 ± 2.43	41 (73.21)	7.92 ± 1.45	7.18 ± 1.66
Observation	58	6.31 ± 1.57	5.94 ± 1.19	55 (94.83)	9.59 ± 1.26	9.55 ± 1.37
*t/χ* ^*2*^		−3.593	−4.601	10.010	6.528	8.305
*P*		0.001	0.000	0.002	0.000	0.000

**Table 5:** Comparison of writing error rates of nursing records [*n* (%)].

Group	*n*	Frequency mismatch	Time not recorded	Clinical practice inconsistencies	Difficulty embodying specialty characteristics	Typos and misrepresentations	Total
Control	56	1 (1.79)	1 (1.79)	2 (3.57)	3 (5.36)	3 (5.36)	10 (17.86)
Observation	58	0 (0.00)	0 (0.00)	0 (0.00)	1 (1.72)	1 (1.72)	2 (3.45)
*χ* ^*2*^							6.281
*P*							0.012

## Data Availability

The data can be accessed upon request from corresponding author.
